# The Development and Acceptability of a Psychology‐Based Intervention for Debilitating Symptom Complexes Attributed to Ticks

**DOI:** 10.1111/hex.70634

**Published:** 2026-03-12

**Authors:** Anita L. Dharan, Valerie M. Z. Yap, Richard A. Kanaan, Georgina Oliver, Yen D. Y. Sim, Sabine Braat, Georgia Cotter, Mary Lou Chatterton, Cathrine Mihalopoulos, Sarah J. Wilson, Trudie Chalder

**Affiliations:** ^1^ Department of Psychiatry University of Melbourne, Austin Health Heidelberg Victoria Australia; ^2^ Centre for Epidemiology and Biostatistics, Melbourne School of Population and Global Health The University of Melbourne Victoria Australia; ^3^ MISCH (Methods and Implementation Support for Clinical and Health) Research Hub, Faculty of Medicine, Dentistry and Health Sciences The University of Melbourne Victoria Australia; ^4^ Monash University Health Economics Group, School of Public Health and Preventive Medicine Monash University Victoria Australia; ^5^ Melbourne School of Psychological Sciences The University of Melbourne Victoria Australia; ^6^ Victorian Collaborative Centre for Mental Health and Wellbeing Victoria Australia; ^7^ Department of Psychological Medicine, Institute of Psychiatry, Psychology and Neuroscience King's College London London UK

## Abstract

**Background:**

Debilitating Symptom Complexes Attributed to Ticks (DSCATT) is a chronic, debilitating illness associated with tick bites in Australia. DSCATT is of unknown aetiology, can impact emotional well‐being and has no recognised treatments.

**Objective:**

The development and piloting of a novel psychotherapeutic adjunctive intervention for DSCATT that aimed to increase daily functioning, improve quality of life and reduce the impact of symptoms in people with DSCATT.

**Methods:**

This is a single‐site, intervention development and acceptability study. The intervention was developed iteratively according to a Human‐Centred Design (HCD) approach and manualised across four phases, with input from end users at each phase: qualitative interviews, development of the intervention, piloting, and revising and refining the intervention. Acceptability of the prototype intervention was evaluated through thematic template analysis of exit interviews. Self‐report measures were completed before and after intervention delivery.

**Results:**

Following qualitative interviews with 13 participants (11 females and 2 males; aged 35–70 years), the intervention was informed by an Acceptance and Commitment Therapy (ACT) model interwoven with cognitive and behavioural strategies that targeted DSCATT‐specific difficulties. The manualised intervention consisted of 12 1‐h weekly individual sessions, delivered by psychologists via Telehealth (video call or telephone). Modules addressed the six core psychological processes of ACT, alongside DSCATT‐specific modules addressing cognitive function, sleep and social relationships. Pilot testing and follow‐up interviews were conducted in a separate sample of six individuals with DSCATT (all females; aged 46–71 years). All participants reported that the approach benefited their emotional well‐being and overall health and would recommend it to others with DSCATT.

**Conclusions:**

This is a novel and theoretically driven psychotherapeutic intervention for DSCATT, co‐produced with patient involvement across four phases. Pilot testing suggested the manualised intervention was feasible and acceptable, supporting future evaluation of feasibility and treatment outcomes with randomised controlled trials.

**Patient or Public Contribution:**

This study involved the engagement and participation of individuals with DSCATT across four stages of the project, according to an HCD approach—the choice of the intervention; the development of the intervention; the piloting of the intervention, and the assessment of the intervention.

**Trial Registration:**

The pilot study component of this project was prospectively registered on the Australian and New Zealand Clinical Trial Registry (ANZCTR); trial ID: ACTRN12621001032842.

## 1 Introduction

Lyme disease is the most common tick‐borne illness worldwide, with its causative agent, the bacterium *Borrelia burgdorferi*, found in 14.5% of the global population [1]. In Australia, there are individuals with a constellation of multi‐system, chronic, Lyme‐like symptoms associated with tick bites who often identify as having Lyme disease. However, they are not recognised by the Australian Government as having the disease, as *Borrelia burgdorferi* has not been definitively identified in Australian ticks [2], and tests from accredited laboratories typically return negative results for Lyme disease. The government termed this condition Debilitating Symptom Complexes Attributed to Ticks (DSCATT) [3].

While the aetiology of DSCATT remains unclear, the persistent and debilitating nature of the illness is evident. People with DSCATT have on average five to six symptoms, with a median symptom duration of 10 years [4]. Most will try multiple antibiotics, sometimes for years, and illness burden is high, with 31% of patients bedbound or unable to walk, and 59% unable to work or study [5]. They describe frustration at the lack of diagnostic clarity and support from doctors, driving them to seek alternative treatments overseas [6].

The multidimensional impact of DSCATT includes adverse effects on an individual's emotional and social well‐being [6], pointing to the need for a holistic treatment approach that extends beyond a biomedical model of illness [7]. There is currently no research into a pharmacological intervention for DSCATT, likely impeded by the lack of clarity over its aetiology. However, studies have shown that non‐pharmacological interventions informed by psychotherapeutic principles of cognitive and behavioural change have helped people with other chronic health conditions manage the extent to which their symptoms interfere with their daily functioning, quality of life and psychosocial well‐being [7, 8, 9, 10, 11, 12]. If applied to people with DSCATT, such interventions would recognise that interactions between cognitive‐behavioural and physiological factors can contribute to the persistent nature of symptoms [13, 14], without endorsing a specific aetiology for the illness.

The aim of the current study was to develop and pilot a novel psychotherapeutic adjunctive intervention for DSCATT that aimed to increase daily functioning, improve quality of life and reduce the impact of symptoms in people with DSCATT. This article describes the conception, development, feasibility and acceptability of the intervention, guided by a Human‐Centred Design (HCD) approach.

## 2 Materials and Methods

### 2.1 Design

**Figure 1:**
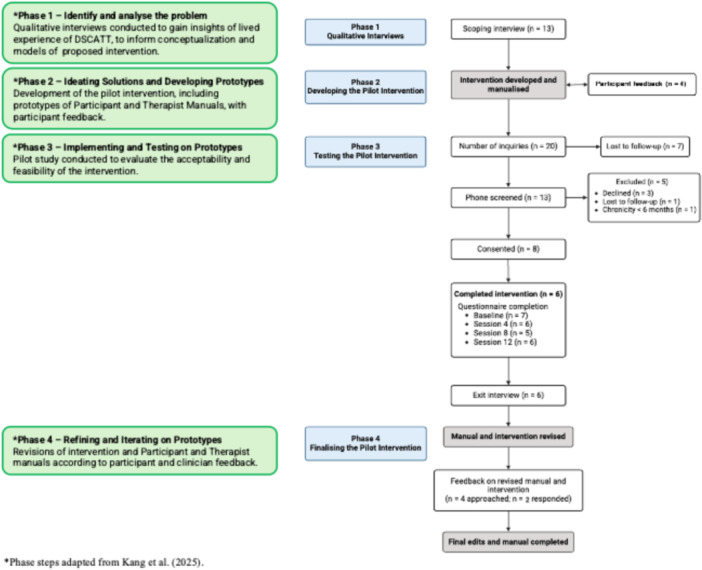
Consort diagram and overview of study aims for each phase, presented in alignment with a Human‐Centred Design approach.

The intervention was developed iteratively over four phases, with consumer engagement occurring in each phase (Figure 1). The development of the intervention followed an HCD approach. This is increasingly being applied to complex healthcare challenges [15], given its emphasis on an empathy‐driven understanding of health needs, and collaborative, iterative development of solutions alongside end‐users [16].

Phases were guided by steps commonly conceptualised within an HCD framework, namely, identifying and analysing problems; ideating solutions and developing prototypes and testing, refining and iterating prototypes [17] (see Figure 1). In line with these steps, phases of the design process included the following: (1) conducting qualitative interviews to explore views on using psychotherapeutic approaches to minimise the impact of DSCATT on daily functioning, quality of life and emotional and social well‐being; (2) the development of a theoretically driven pilot intervention and prototype of a corresponding therapist and Participant Manual, with feedback from key stakeholders; (3) testing the intervention in a pilot study to determine its feasibility and acceptability, and (4) revising and refining the intervention protocol with input from exit interviews (see Figure 1). Approval was granted by the Austin Health Human Research Ethics Committee (project numbers HREC/62334/Austin‐2020 and HREC/69382/Austin‐2020).

### 2.2 Recruitment

For recruitment purposes, DSCATT was defined as a chronic illness (6 months or longer) attributed to a tick bite which could not be better explained by another medical condition. For Phases 1 and 3, we recruited adults (aged 18 years or older) with English‐language proficiency who identified as having DSCATT to participate in the scoping interviews and pilot intervention. Participants were required to have access to Telehealth facilities (e.g., internet, device with video conferencing software and phone service) for remote participation. Participants were recruited from the Austin Health Infectious Disease Unit (Melbourne, Australia) or via self‐referral.

Following informed consent procedures, candidates for Phase 3 had a Telehealth appointment (video call via Zoom) with the project coordinator to discuss their medical history, current presenting symptoms and disability, and undertake the Mini International Neuropsychiatric Interview (MINI) v7.0.2 [18] for screening purposes. Candidates were deemed ineligible for the pilot study if they (a) met DSM‐5 criteria on the MINI for a current psychotic disorder or drug and alcohol abuse; (b) were using benzodiazepine(s) exceeding the equivalent dose of 10 mg of diazepam per day; (c) were at imminent risk of suicide or harm to self or others; or (d) had a serious or unstable medical condition that may interfere with study participation, as determined by the study's medical monitor.

Purposive and snowball sampling was utilised for Phase 1 (qualitative interviews), as described elsewhere [6]. For Phase 3 (pilot intervention), we aimed to recruit 12 participants to allow for a purposive sample and sufficient qualitative information to meet study objectives.

### 2.3 Procedure

#### 2.3.1 Phase 1: Qualitative Interviews

Preliminary qualitative interviews were conducted with 13 participants to gain insights into the lived experiences of people with DSCATT and explore their views about the role of psychological interventions in minimising the impact of symptoms on daily functioning, psychosocial well‐being and quality of life [6]. The methodology of the interviews and qualitative findings about lived experience are described in detail elsewhere [6]. Participants were also provided with brief descriptions of two common cognitive‐behavioural models that have been used with other chronic illness populations, specifically Cognitive Behavioural Therapy (CBT [19]) and Acceptance and Commitment Therapy (ACT [20]; Supplementary Text 1). They were invited to comment on the potential value of these approaches for minimising the impact of symptoms and to indicate their preference for CBT or ACT. Interviews were audio‐recorded and transcriptions reviewed by two researchers to identify common views about these approaches shared by participants.

#### 2.3.2 Phase 2: Developing the Pilot Intervention

Participants' responses from the qualitative interviews were used to inform the development of a pilot intervention for DSCATT, including the selection of the psychotherapeutic model, and considerations for its content and delivery. The intervention was designed to be administered by a clinician across 12 one‐hour, weekly individual therapy sessions. The intervention was co‐developed by two trial clinicians (psychologists) in collaboration with experts who specialise in treating persistent physical symptoms, including a psychotherapist experienced in CBT and ACT who specialises in treating chronic health conditions, a clinical neuropsychologist who specialises in treating functional neurological disorder and a psychiatrist. It was manualised to standardise the delivery of modules, with flexibility given to the trial clinician to tailor the intervention to address individual needs. A Participant Manual was developed as a companion guide for participants, and a Therapist Manual was developed to outline intervention procedures for the clinician and to suggest topics to cover each session.

Prior to finalising the content of the pilot intervention and manuals, participants from the preliminary qualitative interviews (Phase 1) were reapproached to provide feedback on the proposed aims, rationale, format and topics/skills included in preliminary versions of the manuals. Participants who volunteered to share their views were contacted via telephone. The content of the intervention was described by a clinician, and a discussion was had about the different aspects of the intervention, and suggested changes proposed by the participant.

#### 2.3.3 Phase 3: Testing the Pilot Intervention

##### 2.3.3.1 Intervention Structure

Following Phase 2, a pilot study was conducted to determine the feasibility and acceptability of the intervention in a separate sample of participants with DSCATT. Once screened for eligibility and enrolled, participants were allocated to trial clinicians who had been trained in the primary psychotherapeutic model (ACT). Participants were given the option to attend their therapy sessions in‐person or via Telehealth. If attending via Telehealth, Zoom video conferencing was the preferred modality. Where video calls were not possible (e.g., due to connectivity or technical issues), sessions were conducted via telephone. Participants were encouraged to attend one session per week over 12 consecutive weeks, with flexibility for cancellation or rescheduling as required. Trial clinicians recorded the number of missed appointments, reasons for non‐attendance and duration of each session. Weekly appointment reminders were also sent to participants via email or text message.

Trial clinicians referred to the Therapist Manual for a list of suggested topics to cover in each session, with flexibility to tailor the order of topics across sessions depending on the participant's needs. All participants received a hardcopy of the Participant Manual, and homework tasks were negotiated (e.g., readings, worksheets and audio‐guided exercises) to complete between sessions. Trial clinicians emailed participants a summary of the homework tasks following each session. With the participant's consent, each session was audio‐recorded for trial clinician training and supervision purposes. Trial clinicians received regular supervision by senior clinicians to maintain intervention quality and adherence to the manual.

##### 2.3.3.2 Exit Interview and Feedback Survey

Following completion of their final session, participants were invited to attend a semi‐structured feedback exit interview (Supplementary Text 2) with the project coordinator over Telehealth (Zoom video call). Interviews were audio‐recorded with the participants' consent to enable transcription and thematic analysis of the interview transcripts. Participants were asked about their experience of the intervention, with responses explored using additional open questions and probes. A feedback survey providing quantitative data was also administered verbally by the project coordinator, with participants using 4‐point Likert‐type scales to rate the following items: (1) their overall experience of the approach; (2) its benefits to their physical, emotional and overall well‐being; (3) how inclined they were to recommend it to others with DSCATT, and (4) whether it improved their understanding of DSCATT (see Table 7 for specific questions and Likert scale response options).

##### 2.3.3.3 Treatment Credibility and Outcome Questionnaires

Prior to commencing the intervention (baseline), participants completed the Credibility/Expectancy Questionnaire (CEQ) [21] online via REDCap [22, 23] to rate their initial impressions of the intervention's credibility and potential to improve outcomes in people with DSCATT. The CEQ consists of six items, divided into two sets that corresponded with the participant's cognitive beliefs (Set 1 = what one thinks will happen) and affective beliefs (Set 2 = what one feels will happen) about the intervention. See Table 5 for the list of CEQ items. Participants rated each item on a percentage scale (*0* = *not at all logical/useful/confident* to *100 = very logical/useful/confident*). Items 1–3 and 5 were rescaled to the measure's original 9‐point Likert‐type scale for data analysis.

Participants completed the Patient Global Impressions Severity (PGI‐S) and Improvement (PGI‐I) Scales [24] online to rate their overall impression of symptom severity and improvement at Session 12 relative to baseline. Both scales consist of a single item rated on 4‐ or 7‐point Likert‐type scales (PGI‐S: *1 = absent* to *4 = severe*; and PGI‐I: *1 = very much better* to *7 = very much worse*). Participants also completed additional electronic self‐report questionnaires online at baseline and at Session 4, 8 and 12 to measure a variety of treatment outcomes, including: (a) quality of life; (b) levels of functioning; (c) mood; (d) cognitive and behavioural responses to symptoms; (e) symptom severity and improvement, and (f) resource use. These additional self‐report measures and their associated constructs are listed in Table 1. A detailed description of each measure is presented in Table S1.

**Table 1:** Self‐report questionnaires assessing treatment outcomes for the pilot study and timepoints administered.

Construct	Measure	Timepoint (session)
Presence of tick‐borne illness	Horowitz Multiple Systemic Infectious Disease Syndrome Questionnaire (HMQ)^a^	0, 4, 8, 12
Perceptions/beliefs about the illness	Revised Illness Perception Questionnaire (IPQ‐R)^b^	0
Overall impression of symptoms	Patient Global Impression Scale (PGI), with two items measuring symptom severity (PGI‐S) and symptom improvement from baseline (PGI‐I)^c^	0, 4, 8, 12
Quality of life	Assessment of Quality of Life‐8 Dimensions (AQoL‐8D)^d^	0, 4, 8, 12
Levels of functioning	Work and Social Adjustment Scale (WSAS)^e^	0, 4, 8, 12
Psychological flexibility	Comprehensive Assessment of Acceptance and Commitment Therapy (CompACT)^f^	0, 12
Other cognitive and behavioural processes	Cognitive Behavioural Responses Questionnaire‐Short Version (CPRQ‐S)^g^	0, 12
Depression and anxiety	The Hospital Anxiety and Depression Scale (HADS)^h^	0, 4, 8, 12
Resource use	Purpose‐built health service Resource Use Questionnaire (RUQ)^i^	0, 12

*Note:* Timepoint “0” refers to baseline.

^a^ Citera et al. [25].

^b^ Moss‐Morris et al. [26].

^c^ Guy [24].

^d^ Richardson et al. [27].

^e^ Mundt et al. [28].

^f^ Francis et al. [29].

^g^ Ryan et al. [30].

^h^ Zigmond and Snaith [31].

^i^ The Resource Use Questionnaire was adapted by health economists within the research team from previous Australian trials of mental health interventions and assesses the type, frequency and duration of health service use across diagnostic testing, medication use, outpatient, emergency and inpatient care. Questions also gathered data on lost paid and unpaid work as well as an estimate of productivity while at work with symptoms (presenteeism).

The project coordinator completed the Clinical Global Impressions Severity (CGI‐S) and Improvement (CGI‐I) Scales [24, 32] to provide an external clinician‐report of each participant's symptom severity (CGI‐S) and symptom improvement (CGI‐I) at exit interview relative to baseline (screening). The project coordinator's responses on both scales were based on information provided by the participant during the exit interview and accompanying feedback survey. This was compared to their screening interview, and any change to their overall health was assessed. Both scales consist of a single item rated on a 7‐point Likert‐type scale (CGI‐S: *1 = normal, not at all ill* to *7 = among the most extremely ill patients*; CGI‐I: *1 = very much improved since the initiation of treatment*, to *4 = no change from baseline*, to *7 = very much worse since the initiation of treatment*). They were originally designed to measure the clinician's impression of a patient's post‐treatment changes in psychiatry settings [24]. In this study, both scales were completed based on the project coordinator's overall impression of the participant's general health, rather than mental health status alone, given the multifaceted nature of their symptoms.

##### 2.3.3.4 Safety Reporting

Assessment and recording of adverse events (AEs) was informed by Klatte et al. [33], Herzog et al. [34] and the National Health and Medical Research Council (NHMRC) Safety Monitoring and Reporting guidelines [35]. AEs were defined as worsening of pre‐existing symptoms, diagnosis of a new medical condition and/or emergence of new or recurrent symptoms or injuries. As per the NHMRC, serious AEs (SAEs) were defined as events that resulted in death, were life‐threatening, required hospitalisation or prolongation of existing hospitalisation, resulted in persistent or significant disability or incapacity, or were considered a congenital anomaly or birth defect [35].

AEs identified via participant report or trial therapist observation were documented and reviewed internally by the medical monitor to assess severity, causality to treatment or other study procedures and to provide a recommended course of action where necessary.

#### 2.3.4 Phase 4: Revising and Refining the Intervention

Following the pilot study, different aspects of the intervention were revised in preparation for a feasibility, randomised controlled trial (RCT) of the intervention. Specifically, the Participant Manual content was modified and information added at the discretion of trial clinicians and clinical supervisors, as well as in response to participant feedback from the exit interview. Participants from the pilot study were invited to provide feedback via email about specific modifications to the Participant Manual. Further edits based on this feedback were made accordingly.

Modifications to the intervention duration (e.g., number of sessions) were also made based on the clinical team's impressions and participant feedback from the exit interview. The Therapist Manual was revised accordingly to reflect an updated session plan.

### 2.4 Data Analysis

For Phase 3, the feasibility of the pilot intervention was determined by the proportion of eligible participants who consented to participate (recruitment rate) and those who completed the intervention upon enrolling (retention rate). The acceptability of the pilot intervention was assessed by qualitative analysis of the participants' responses on a post‐intervention exit interview, ratings from the feedback survey administered verbally at the exit interview and ratings from the CEQ [21] administered at baseline.

To describe outcomes of feasibility and acceptability in Phase 3, categorical data (e.g., recruitment and retention rates, ratings on feedback survey at exit interview) were reported using measures of relative frequency (e.g., percentages), whereas data from the CEQ were reported according to median and range. Qualitative data (participant feedback) from the exit interviews were analysed following principles of thematic template analysis [36]. Audio‐recordings of the interviews were transcribed using Zoom and Microsoft Word, with personal identifiers omitted. Transcripts were reviewed for accuracy by the trial clinicians and project coordinator. A hybrid inductive–deductive approach was used to allow themes to be conceptualised from participant views as well as informed by a theoretical framework of acceptability of healthcare interventions [37]. Using an inductive approach, transcripts were first openly coded with NVivo software (V.12) to identify key information related to each participant's experience and their feedback about the intervention. Initial codes were grouped into subthemes based on common features in the participants' responses. Using a deductive approach, subthemes were then assigned to broader a priori themes. The themes corresponded to a theoretical framework that defines the acceptability of healthcare interventions according to seven component constructs [18]: (1) affective attitude; (2) burden; (3) ethicality; (4) intervention coherence; (5) opportunity costs; (6) perceived effectiveness, and (7) self‐efficacy. Subthemes that did not fit the seven themes of acceptability were then reanalysed inductively for commonalities and subsequently collapsed into additional themes. Codes and themes were identified by consensus between four researchers.

## 3 Results

### 3.1 Phase 1: Qualitative Interviews

Among the 13 participants interviewed (11 females and 2 males; age range 35–70 years) [6], there were mixed views about the role of psychological interventions in minimising the impact of DSCATT symptoms on daily functioning, quality of life, and emotional and social well‐being. Participants generally acknowledged the benefits of psychological interventions for supporting their mental health but highlighted that they should not be used as a singular approach for treating the illness. They reasoned that psychological methods alone are unlikely to reduce symptom severity and risk perpetuating the notion that the illness is ‘all in their heads’, invalidating concerns about an underlying organic (e.g., ‘biological’, ‘physiological’) cause for their illness. Additionally, some participants stated that funding should instead be prioritised for research investigating pathogenic mechanisms of the illness and corresponding treatment options. There were no definitive preferences for CBT or ACT, and most participants discussed these psychotherapeutic models interchangeably.

### 3.2 Phase 2: Developing the Pilot Intervention

The 12‐session pilot intervention was informed by findings from the scoping interviews and suggestions from a subset of participants from Phase 1 (*n* = 4; 2 female, 2 male) who volunteered to provide feedback on the proposed intervention. The intervention comprised a hybrid approach combining principles of ACT with traditional behavioural strategies (e.g., activity monitoring and scheduling). ACT was selected as the primary psychotherapeutic model of choice based on its transdiagnostic potential and core principle of promoting psychological flexibility [20, 38]. Psychological flexibility refers to one's ability to adapt to challenges by paying attention to the present, being open to a range of inner experiences (e.g., thoughts, emotions and sensations) and pursuing meaningful activities that align with one's values [20]. A primarily ACT‐based intervention was deemed suitable, given the chronic nature of the illness, uncertainty about its course, heterogeneity of symptoms and psychosocial sequelae experienced by people with DSCATT. Psychoeducation about the biopsychosocial framework of health [7] was also included in the intervention.

Table 2 presents the list of topics that were covered across the 12 sessions. The intervention was organised into three main phases: (1) assessment of presenting problems and introduction to the rationale and model of treatment; (2) active treatment, and (3) preparation for discharge, including a review of progress and plan to manage setbacks post‐treatment. The active treatment phase included key modules consistent with ACT principles (e.g., connecting with values; taking committed action; utilising mindfulness for defusion, contacting the present self‐as‐context; building acceptance) [20, 39]. It also included optional modules to address wider concerns often reported by participants (e.g., self‐compassion, sleep, memory/brain fog and social relationships). Content related to managing pain and difficult emotions, living with uncertainty, destigmatising the psychological impacts of illness, exercise, improving sleep and managing social relationships was suggested by participants who provided feedback. They endorsed the potential utility of this intervention approach and maintained that biological attributions to the illness should not be dismissed.

**Table 2:** Pilot intervention program for DSCATT: Session overview.

Session number	Key topics
*Phase 1: Introduction and assessment*	
1	Introduce the program (aims, session structure) Rationale for intervention (biopsychosocial framework; transdiagnostic, patient‐centred approach) Introduce ACT model Assessment/history‐taking
2	Review rationale for treatment Initial case formulation (‘choice point’^a^) Discuss values Discuss monitoring of daily routine over the week
*Phase 2: Active treatment*	
3	Continue values work Review daily routine monitoring (baseline levels of activity/rest) Identify targets for behavioural intervention Establish simple activity schedule Introduce mindfulness
4	Review simple activity schedule (discuss progress/barriers) Introduce SMART^b^ goal setting (values‐guided) Establish weekly committed action plan Continue mindfulness work (experiential exercises)
5	Review mindfulness techniques Review weekly committed action plan (discuss progress/barriers) Establish new weekly committed action plan Introduce acceptance (dropping the struggle with uncertainty and discomfort)
6–10	Introduce self‐compassion *Optional modules:* Improving sleep Managing thinking and memory difficulties Managing relationships *Modules to repeat (if needed):* Review treatment rationale Review values and workability of behaviour Establish weekly committed action plan for SMART goals Practise mindfulness skills (including skills to build acceptance and self‐compassion)
*Phase 3: Discharge planning*	
11–12	Review case formulation and progress Review key modules Discuss goal‐setting post‐intervention Discuss managing setbacks

*Note:* The order of topics could be rearranged across sessions based on clinical judgement.

Abbreviations: ACT = Acceptance and Commitment Therapy, DSCATT = Debilitating Symptom Complexes Attributed to Ticks.

^a^ Harris [39].

^b^ SMART goals refer to goals that are specific, motivated by values, adaptable, realistic and time‐framed.

The corresponding Participant Manual consisted of 13 chapters (Table 3), inclusive of worksheets and additional exercises to encourage self‐reflection and enhance skills‐training of topics covered in the session. It included case examples of fictional characters with DSCATT and used person‐centred language.

**Table 3:** Overview of Participant Manual for pilot intervention.

Chapter	Title	Objectives
1	Background	Introduce the program, biopsychosocial approach to healthcare, cognitive and behavioural processing of symptoms
2	Model of therapy	Introduce ACT (e.g., ‘workability’, ‘choice point’ tool^a^)
3	Daily routine	Monitor baseline level of activity and rest
4	Increasing levels of activity	Establish a simple activity schedule for behavioural activation
5	Values	Identify values, evaluate connectedness to values
6	Goals	Establish SMART^b^ goals and action plans
7	Unhooking	Explore fusion with difficult inner experiences, introduce mindfulness techniques (e.g., strategies for defusion, staying present and self‐as‐context)
8	Living with uncertainty	Explore avoidance vs. willingness (acceptance), dropping the struggle with uncertainty and physical discomfort, introduce strategies to build acceptance
9	Self‐compassion	Explore self‐critical thoughts, use strategies to develop self‐compassion
10	Improving sleep	Monitor sleep patterns, use strategies to improve quality of sleep
11	Managing thinking and memory difficulties	Provide information about attention, learning and memory; impact of stress on thinking skills, introduce compensatory strategies
12	Managing relationships	Provide tips to improve quality of relationships, manage conflict, set boundaries
13	Wrapping up	Provide summary of the program, review tips to maintain progress and manage setbacks

Abbreviation: ACT = Acceptance and Commitment Therapy.

^a^ Harris [39].

^b^ SMART goals refer to goals that are specific, motivated by values, adaptable, realistic and time‐framed.

### 3.3 Phase 3: Testing the Pilot Intervention

#### 3.3.1 Feasibility

Recruitment and data collection occurred between September 2021 and June 2022. Twenty individuals enquired about the pilot study via email or phone. Thirteen responded to further contact and were screened for eligibility. Twelve individuals were deemed eligible, and eight consented to participate (67% recruitment rate). The other four declined to participate or were lost to follow‐up (Figure 1).

Seven participants commenced the intervention (see Table 4 for demographics), and one was unable to commence due to health reasons. Of the seven, one elected to withdraw after the first session. The remaining six (86% retention rate) completed all 12 sessions and attended the exit interview. Four participants completed 100% of the self‐report questionnaires across all time points.

**Table 4:** Demographic information for all consenting participants in the pilot intervention (*N* = 8).

Demographic	*n* (%)
*Sex*	
Female	7 (87%)
Male	1 (13%)
*Age*	
Median	52
Range	32–71
*Residence*	
New South Wales	2 (25%)
Queensland	2 (25%)
Victoria	3 (37%)
Western Australia	1 (13%)
*DSCATT history*	
Illness duration in years—median (range)	9 (3–21)
Observed tick bite/s	5 (63%)
Experienced a tick bite, erythema migraines (‘bullseye’ rash), or an undefined rash, followed by flu‐like symptoms	5 (63%)
*Employment status*	
Not in paid employment	1 (13%)
Part‐time paid work	3 (37%)
Full‐time paid work	4 (50%)

Abbreviation: DSCATT = Debilitating Symptom Complexes Attributed to Ticks.

Participants completed the intervention over a median span of 13 weeks (range = 11–24 weeks), with a median period of 7 days (range = 6–27 days) between sessions. The median duration of sessions was 74 min (range = 57–140 min). All sessions were delivered via Telehealth (97% [71/73] video call; 3% [2/73] phone call). Eight appointments were missed and subsequently rescheduled across four participants, with reasons for non‐attendance including illness, scheduling conflicts and family/carer commitments.

#### 3.3.2 Safety Reporting

Twenty AEs were reported across six participants, with most constituting the emergence of new symptoms and the flare‐up or worsening of existing symptoms (Table S2). Of the 20 events, one was classified as an SAE, characterised by a hospitalisation for Functional Neurological Disorder symptoms. All AEs were deemed by the medical monitor to be ‘unrelated’ or ‘unlikely related’ to the intervention, except for two (irritability, self‐harming urges) across two participants that were deemed ‘possibly related’.

#### 3.3.3 Acceptability: Responses on the CEQ

Median scores for each CEQ item are presented in Table 5. Prior to starting the intervention (baseline), participants indicated some level of belief that the intervention would be logical and useful for improving their symptoms. There was a trend for a higher level of cognitive belief (Item 4; median = 77.5% [range = 10–100]) compared to affective belief regarding their expectation for symptom improvement post‐treatment (Item 6; median = 55.0% [range = 10–92]).

**Table 5:** Median and range of participant ratings on the CEQ at baseline.

Item	*n*	Median (IQR)	Range
*Set 1: Cognitive beliefs*			
At this point, how logical does the therapy offered to you seem?^a^	6	7.6 (5.6–8.1)	5.0–9.0
At this point, how successful do you think this treatment will be in reducing your symptoms?^a^	5	7.4 (5.4–7.4)	5.0–7.6
How confident would you be in recommending this treatment to a friend who experiences similar problems?^a^	5	7.2 (5.0–7.4)	5.0–7.4
By the end of the therapy period, how much improvement in your symptoms do you think will occur?^b^	6	77.5% (65.3–83.0)	10.0–100.0
*Set 2: Affective beliefs*			
At this point, how much do you really feel that the therapy will help you reduce your symptoms?^c^	6	6.2 (5.1–7.6)	1.8–8.2
By the end of the therapy period, how much improvement in your symptoms do you really feel will occur?^b^	6	55.0% (42.5–71.3)	10.0–92.0

Abbreviations: CEQ = Credibility/Expectation Questionnaire, IQR = interquartile range.

^a^ 9‐point Likert scale (*1 = not at all logical/useful/confident*, *5 = somewhat logical/useful/confident*, *9 = very logical/useful/confident*).

^b^ Ratings were made on a scale of 0%–100%.

^c^ 9‐point Likert scale (1 = not at all, 5 = somewhat, 9 = very much).

#### 3.3.4 Acceptability: Themes From Exit Interview

Qualitative analyses of responses from the exit interview revealed themes corresponding to six of the seven key constructs of Sekhon et al.'s [37] framework for the acceptability of healthcare interventions. No theme emerged corresponding to the construct of ‘opportunity costs’ (i.e., degree to which one must compromise what they value to participate in the intervention). Themes related to suggestions for improvement are also described below. Quotes for each theme are presented in Table 6.

**Table 6:** Selected quotes from the exit interview corresponding to themes of acceptability.

Theme	Quote
Affective attitude	*Positive experience despite initial reservations* I was a bit anxious and nervous coming in…I though, what's this all going to be about, because you know there's that preconceived idea…Because you might have people avoid it because they think it's all going to be, well ‘you've got this pain, because your parents were bad to you’…And we don't know what we're coming into. So, I took a leap of faith and I'm so glad I did. (P890) *Positive feedback about strategies and resources* So, the techniques have been wonderful for me…I've got my favourites, so my absolute favourite is Leaves on the Stream. (P345) *Positive relationship with trial clinician* Partly that's to do with [clinician] and the connection…But also that [clinician] listened and was empathetic and heard and adapted, rather than sticking to the plan…That maturity and confidence of [clinician]…Just a big thanks to the whole team, as I said, particularly [clinician] for her patience, understanding, adaptability, and relationship building as well. (P765) *Recommending the program to others* Yeah from anything from mental illness to physical illness to anything, it really is a fantastic program across the board, I think, for anybody to do…It was really helpful, and I think I said to [clinician] at one stage, I think this would be a really great program to do with just kids at secondary school or kids at primary school, like Grade 5 and 6. (P321)
Burden	*Impact of symptoms* Some weeks I wasn't feeling very well, and I couldn't put things into practice the way I would've liked to. (P123) *Session length* Sometimes you get to the point where you just start opening up and start getting into a lot of things, and it's the end of the session…I think if you offered an hour, then in the background, you may be allowed it to run to an hour and a half, it would allow that stuff to come out. (P567) Half an hour is beautiful. If I did notice we did a couple things up to the hour, I did, was looking at my clock a couple of times and I had lost some interest and had to bring myself back. But those half hours, sometimes if we're just aiming for the half hour, I was more than happy for it to go over. If I felt I sometimes had the one hour, it was like, ugh. (P890)
Ethicality	*‘Fit’ of the program* It was a good fit; it was a good fit for me. (P345) In fact, reading the book [manual], everything, it was interesting reading this too, I was like wow you, you know me…I actually would go, tick, oh my gosh, yes…Really spoke to me, except Chapter Eight [‘Living with Uncertainty’]…All I know is that Chapter Eight, I felt really uncomfortable with. (P890)
Coherence	*Relevance of the program and manual* And the best thing I found out about it, about it, was the anchoring. The anchoring exercises. I find that really good. And the beachball analogy…You can't hold the ball underwater and you get so tired and fatigued…You just get tired by just trying to deal with the thoughts and the feelings all day long. Whereas if you just let them up, do the anchoring exercises, acknowledge that they're there…I just like the way it put it across really easy…It basically allows you to just acknowledge them [thoughts] rather than keep fighting them. (P123) The book was really good and having the book was great…And whoever did the pictures for the book, they were really good…Yeah, as you go through it and even reading it, it just makes sense and it's not heavy terrain so you're not thinking, ‘I don't understand this’, you know, it's, it's quit light to read and easy to read… And then, with the pictures on top of that, you know, to reinforce. What you've just read, it is really simple. It's a terrific manual. (P321)
Perceived effectiveness	*General support* It's given me some tools and techniques that I will not let this, I've always said, I will not let this disease, call it whatever you want, beat me. I will not let it beat me. (P345) *Psychological flexibility* I'm a bit more accepting…It's just you have to endure and get past it…Learning to live with it…I think it's just over a period of time recognising that the disease may not go, and just learning to live with it…The acceptance, that you may have this for the rest of your life, we don't know…I possibly may go into remission, and I may not, but what am I going to do in the meantime? (P123) *Committed action* Just trying to pace myself a little bit more and say it's okay to, you know, don't beat yourself up if you, if you do things in little pieces…Because it gives you more energy for the next day…So, I have found that ‘boom bust’ really helpful…Because I did less, heavy intensity, like I had tried to break it down. And so that's when I'm thinking I got my benefit…So the overall symptoms didn't change, but those crash days were less, because of, just trying to do this less ‘boom bust’. (P890)
Self‐efficacy	*Adapting the program to facilitate engagement* One week was cancelled ‘cause we just couldn't do anything else. When we got together the next week, she [clinician] was able to adapt and change the program to fit in the bits that helped me at that time…That was absolutely marvellous because I've thought, for sure, that I would more than likely be out of the study because of what had happened [health event]. (P345) *Engaging in the acute phase of illness* So if I had of been doing this program when I got first got sick, there's no way possible…I wouldn't have had the concentration to listen and participate…Wouldn't have had the concentration or the ability to do any of the homework stuff…I just don't think it would work because it's just too, too involved in daily life, whereas when you've got Lyme, there is no daily life. (P321)
Suggestions for improvement	*Extending the program* It felt like it tried to do too much in too short a time. But I wouldn't know what to remove, because I think it was all important. And so therefore, the only way I could think of resolving that would be to extend it to maybe 16 weeks or 15, 16 weeks…But in 12 weeks, that's far too much in 12 weeks…And so you know, I think it's a bigger program…But the more holistic stuff‐‐sleeping, relationships, conversations‐‐on top of some of those immediate things, I felt like I was just beginning to get my head around them when it ended…So therefore, I feel like I was just beginning to get this benefit at the end. (P765) *Holistic care* It would have been very helpful to have the whole package…So I think that's what I'm saying with this holistic approach. I think it's all important. What I'm fearful of is that your domain and then an infectious disease domain as separate, and nobody talks to each other to come up with a nice all‐round program…Because in order for yours to be successful, the physical side needs to be addressed, where people can get some relief. (P123)

##### 3.3.4.1 Affective Attitude

‘Affective attitude’ refers to sentiments about the intervention. All participants reported having a positive experience, including two who initially had reservations about participating due to uncertainty about what the intervention involved and apprehension that their illness may be misunderstood. Overall, participants valued the strategies introduced in session (e.g., mindfulness, committed action) and resources provided (e.g., Participant Manual, worksheets and audio guides). Participants positively described their therapeutic relationship with their trial clinician. They were pleased with their clinician's ability to tailor the intervention to suit their needs and felt that their concerns were acknowledged and validated. Further, participants valued the transdiagnostic nature of the intervention and expressed that they would recommend it to others, including other clinical and non‐clinical populations more broadly. Two participants also reported that they preferred this approach to other pain management programs that they had previously participated in.

##### 3.3.4.2 Burden

‘Burden’ refers to the perceived amount of effort required to participate in the intervention, including time commitment, ease of accessibility and cognitive effort involved. Participants reported that the extent of burden depended on the severity and fluctuation of their symptoms, with greater difficulty attending sessions, doing homework or practicing skills during a flare‐up. Overall, they did not raise significant concerns about the volume of homework tasks (e.g., worksheets, readings), although one participant reported that readings for the first session were ‘too much’ when given with other study tasks (e.g., baseline questionnaires).

Some participants reported that the questionnaires were time‐consuming and effortful to complete, although the option of doing them in sections (e.g., saving responses and resuming later) and being informed about the expected duration were noted to be helpful. Participants had mixed views about the intervention session length, with some preferring additional and longer sessions (> 1 h) to cover and consolidate the material and others finding 1‐h sessions sufficient or preferring shorter sessions. One participant noted the convenience of attending the sessions via Telehealth.

##### 3.3.4.3 Ethicality

‘Ethicality’ describes participant views on whether the intervention was a good fit with their value system. Given the positive review by participants, the intervention as a whole appeared to align with the group's values. However, certain techniques and modules did not resonate with some participants. Two participants disliked a specific mindfulness strategy (‘Dropping Anchor’ [39]), with one noting that the audio guide they received for this exercise was too generic (not tailored to their situation), and the other noting that the technique itself was counterproductive as it magnified their symptoms further. Additionally, one participant disagreed with the notion that ‘acceptance/willingness’ could help individuals with DSCATT to manage their physical discomfort (Chapter 8, Participant Manual), especially in the acute stage of illness.

##### 3.3.4.4 Coherence

*‘*Coherence’ refers to the extent to which participants understand the rationale for intervention and how its different components (e.g., strategies, resources) work to achieve its aim. Participants reported that the intervention and Participant Manual were relevant to their experience and provided practical skills that they could apply. They noted that the manual's content was well‐written, and they found the inclusion of visual prompts (e.g., diagrams, images) and metaphors for therapeutic processes useful for understanding key concepts.

Regarding the self‐report questionnaires, participants reported that they did not always capture the nuances of symptom fluctuations or symptom experiences. Some participants also found the wording of questions to be unclear and were confused by changes in the timeframe of reference between questionnaires (e.g., rating items based on the past 1 month vs. no timeframe stated in instructions).

##### 3.3.4.5 Perceived Effectiveness

‘Perceived effectiveness’ describes participant views on the utility and effectiveness of the intervention. Participants acknowledged that the intervention helped them to manage their illness more effectively and reported various benefits which aligned with processes of psychological flexibility. Mindfulness strategies helped them to be more present, acknowledge and observe their thoughts/feelings, create some distance from their discomfort and exercise self‐compassion. Some participants also reported feeling more relaxed after practising mindfulness. In addition, participants acknowledged that cultivating acceptance (e.g., dropping the struggle with illness) could help them to move forward and engage with other meaningful aspects of life.

Participants benefited from taking committed action in the service of their values, for example, by pacing their levels of activity and rest (e.g., minimising ‘boom and bust’ behaviours) and breaking down larger tasks into smaller steps. Simplifying goals into achievable action plans enabled them to connect with meaningful activities, even if activities were modified to suit their current levels of functioning rather than premorbid levels. One participant also reported that they benefited from the practical strategies provided in the optional module for improving sleep. On balance, participants did not generally report a reduction in symptoms.

##### 3.3.4.6 Self‐Efficacy

‘Self‐efficacy’ refers to the participant's confidence in their ability to engage with the intervention. Participants found that the flexible nature of the intervention fostered their engagement, especially during a flare‐up or fluctuation of symptoms. They valued the clinicians' ability to adapt the session plan around their current needs and address what was relevant. Participants also reported that the summary of homework tasks and appointment/homework reminders they received between sessions were helpful for staying on track. However, participants reported that their engagement would have been limited if they were still acutely unwell or new to living with DSCATT, due to factors like physical discomfort, fatigue and impaired concentration.

##### 3.3.4.7 Suggestions for Improvement

Participants suggested that the intervention should be extended to allow more time to discuss their illness experience and consolidate or review the content covered. Specific suggestions included allocating extra time for individual sessions (> 1 h), increasing the number of sessions (> 12 sessions), and adding follow‐up sessions (post‐intervention) to revise the skills taught previously and assess whether progress is maintained. One participant suggested that the intervention be integrated with other health services to promote a multidisciplinary approach for holistic patient care. Other suggestions included providing more information about what the intervention involves (at recruitment), recording audio guides for mindfulness exercises in the clinician's voice and collaborating with the wider patient group to inform study design.

#### 3.3.5 Acceptability: Responses on Feedback Survey

Data from the feedback survey (included as part of the exit interview) are presented in Table 7. All participants reported having a positive experience with the intervention overall and said they would recommend it to other individuals with DSCATT. All participants reported that the intervention had improved their overall health and emotional well‐being, although they had mixed responses about its benefits for their physical health, with 33% (*n* = 2) reporting no benefits. Participants also provided mixed responses about the intervention's role in improving their understanding of DSCATT, with the majority (67%, *n* = 4) reporting no or minimal benefits.

**Table 7:** Frequency of ratings on each Likert‐scale option per questions from the feedback survey administered at the exit interview.

Questions	Rating	*n*
Overall, how would you rate your experience of the treatment you got at the DSCATT Pilot Study?	Excellent	5
	Good	1
	Fair	0
	Bad	0
Do you feel that you have benefited from this treatment in terms of your physical health?^a^	I benefited a lot	2
	I benefited a bit	1
	I did not really benefit at all	2
	It made me feel worse	0
Do you feel that you have benefited from the treatment in terms of your emotional well‐being?	I benefited a lot	3
	I benefited a bit	3
	I did not really benefit at all	0
	It made me feel worse	0
Do you feel that you have benefited from the treatment in terms of your overall health?	I benefited a lot	4
	I benefited a bit	2
	I did not really benefit at all	0
	It made me feel worse	0
Do you feel that you have benefited from the treatment in terms of your understanding of DSCATT?	Definitely have	2
	Probably have	0
	Probably not	2
	Definitely not	2
Would you recommend a treatment like the one you had to someone who was suffering from DSCATT?	Definitely would	5
	Probably would	1
	Probably not	0
	Definitely not	0

^a^ One participant did not respond.

#### 3.3.6 Treatment Outcome Questionnaires

Scores from the PGI‐I and CGI‐I scales were evaluated at the individual level to assess perceived post‐treatment improvement for each participant based on self‐report and clinician‐report, respectively (Table 8). On the PGI‐I, 50% of participants (*n* = 3) rated some degree of symptom improvement at Session 12 relative to baseline. On the CGI‐I, 50% of participants (*n* = 3) were rated by the project coordinator to demonstrate some degree of improvement for their general health at the exit interview relative to baseline.

**Table 8:** Frequency of ratings on each Likert‐scale option from the Patient Global Impression of Improvement (PGI‐I) and Clinical Global Impression of Improvement (CGI‐I).

Questions	Rating	*n*
PGI‐I: Please select the option that best describes how your symptoms are now, compared with how they were before you started this study	Very much worse	1
	Much worse	1
	A little worse	0
	No change	1
	A little better	2
	Much better	1
	Very much better	0
CGI‐I: Rate total improvement (from baseline) whether or not, in your judgement, it is due entirely to the treatment	Very much worse	0
	Much worse	1
	Minimally worse	0
	No change from baseline	2
	Minimally improved	2
	Much improved	1
	Very much improved	0

Median scores per timepoint for the PGI and CGI scales (including PGI‐S and CGI‐S), and full suite of additional self‐report questionnaires, are presented in Table S3. For all questionnaires, there was no consistent trend for improved treatment outcomes across timepoints at a group level. Median scores at Session 12 (end of intervention) were comparable to scores at baseline.

Frequency data from the purpose‐built Resource Use Questionnaire (RUQ) are reported in Table S4, indicating the rate of service use (e.g., health services, hospital attendance and diagnostic tests) and missed education/work at baseline and Session 12.

### 3.4 Phase 4: Revising and Refining the Intervention

#### 3.4.1 Revising the Participant Manual

Upon completion of the pilot phase, the Participant Manual was revised and extended. Revisions were made based on participant feedback from the exit interview (e.g., value of extra sessions, clarification on managing physical symptoms with acceptance) and input from senior and trial clinicians, who drew on their expertise and therapeutic insights from working with the participants to identify additional elements that could benefit individuals with DSCATT. The final manual consisted of 14 chapters, with a new chapter dedicated to building a physiological rationale for the treatment approach (Chapter 2: ‘The Experience of Symptoms’), which was not previously covered in detail. The chapter opens with a description of ‘health’ adopted by the study, specifically as a holistic approach addressing not just the illness itself but also its impact on the person's mental, social and physical well‐being. It then addresses the brain's role in regulating the physiological, cognitive and behavioural processes that contribute to the maintenance of symptoms. Other key topics in the chapter include the following: (a) the adaptive role of ‘sickness behaviours’ in the acute phase of the illness which may influence behavioural responses in chronic stages, (b) cognitive processes (e.g., ‘schemas’) for interpreting illness‐related information, (c) the functional role of emotions and (d) the neurobiology of stress and its adverse effects on health. Information about the immune system and physiological pathways involved in stress (e.g., autonomic nervous system, hypothalamic‐pituitary axis) were also added.

The Participant Manual was also revised to provide more information about the following topics in other chapters: (a) the ‘boom and bust cycle’ and pacing levels of activity and rest [40, 41], (b) the ‘observing self’ [39] and additional metaphors for mindfulness and shifting attention and (c) the ‘three circles model of emotion’ [42] for regulating stress with self‐compassion. Further, based on specific feedback from the exit interview, content about building willingness (acceptance) to manage physical discomfort was revised to promote its application in the chronic rather than acute stages of illness. Additional information was also provided to explain the rationale for using acceptance as a strategy to target the cognitive and behavioural processes that perpetuate the struggle with physical symptoms.

#### 3.4.2 Participant Feedback on the Revised Manual

Four of the six participants who completed the pilot study were invited to read a preliminary version of the revised manual. All expressed interest in providing feedback on the modifications, though only two participants ultimately did so. Feedback was requested specifically on the new chapter (Chapter 2) and on the revised content on building willingness to manage physical discomfort. The participant's feedback was generally positive, and their suggestions to further clarify certain concepts were considered and incorporated into the final version of the manual where appropriate.

#### 3.4.3 Revising the Intervention Structure

Based on participant feedback from the exit interview and the average duration of sessions in the pilot phase (i.e., > 60 min), the clinical team decided to extend the intervention from 12 to 16 sessions for the planned RCT of the intervention. While each session of the RCT intervention would remain at 60 min duration, the first session would be extended to 90 min to allow additional time for history‐taking and building engagement. The Telehealth format would be maintained, although participants could still opt to have face‐to‐face sessions if suitable. The Therapist Manual was revised to accommodate these changes, including an updated session plan with suggested topics to cover in each session of the RCT intervention.

## 4 Discussion

This study describes the development, feasibility and acceptability of a 12‐session ACT‐informed psychotherapeutic pilot intervention designed to improve daily functioning, enhance quality of life and reduce the impact of symptoms in people with DSCATT. The intervention was designed to promote psychological flexibility and values‐based living despite persistent and debilitating symptoms. The intervention was conceptualised, developed and piloted iteratively over four phases with participant feedback throughout, aligning with an HCD approach.

Recruitment for the pilot study was challenging, and the sample size was small (*n* = 6), which warrants a cautious interpretation of findings. Nonetheless, pilot testing suggested that the intervention was both feasible and acceptable. Session attendance and questionnaire completion rates were high, suggesting that engagement demands were not overly burdensome for the patient cohort. Global ratings of the intervention were positive, with five of the six participants describing their experience as ‘excellent’. All participants reported improvements in their overall health and emotional well‐being and endorsed that they would recommend the program to others with DSCATT. Although the small sample precludes meaningful evaluation of the intervention effectiveness, three participants reported some improvements in perceived illness severity following the program. Overall, these findings support the further refinement of the intervention, informed by participant feedback and clinical judgement (e.g., extending the intervention to 16 sessions, updating the Participant Manual). This will provide a foundation for the subsequent evaluation of feasibility and treatment outcomes in a suitably powered RCT.

This was a novel intervention, developed to address the lack of evidence‐based treatments for people with DSCATT. One of the strengths of the study was that the intervention was developed iteratively, with emphasis on co‐development and consumer engagement throughout all phases, in accordance with HCD [16] and NHMRC guidelines [43]. Further, we evaluated the acceptability of the intervention using a defined theoretical framework for the acceptability of healthcare interventions. This allowed for a more robust evaluation of acceptability, which will better inform future feasibility and efficacy studies. An additional strength was that the model underpinning the intervention approach was theoretically grounded, based on a growing body of literature on the effectiveness of psychological interventions to reduce symptom‐related disability in other chronic and medically unexplained conditions [44, 45, 46]. Our intervention was based on similar approaches developed for persistent physical symptoms [47]. This suggests its benefits could extend to other chronic health conditions, highlighting the transdiagnostic potential and aetiologically neutral nature of the intervention approach.

There are several limitations to the study. Due to the small sample size and the lack of a control group, effectiveness could not be assessed and mediators of treatment effect could not be meaningfully evaluated. There were no discernible trends noted on outcome scales. As a result, conclusions regarding the impact of the intervention on specific outcome measures remain unclear. Beyond the limitations of the small sample size, participants had a longstanding illness (median duration of 9 years). Hence, the relatively brief intervention may have been insufficient to produce clinically relevant, quantitative improvements in broad functional domains such as daily functioning, quality of life and emotional or social well‐being. This is consistent with evidence that cognitive‐behavioural interventions for comparable conditions (e.g., chronic fatigue syndrome) typically span a median of 26 weeks [48]. Addressing these limitations, the next step will be to conduct an adequately powered RCT to establish the efficacy of the intervention, contributing to the urgent need for evidence‐based interventions for DSCATT.

## Author Contributions

**Anita L. Dharan:** formal analysis, investigation, visualization, original draft preparation, writing – review and editing. **Valerie M. Z. Yap:** formal analysis, investigation, visualization, writing – original draft preparation, writing – review and editing. **Richard A. Kanaan:** conceptualization, funding acquisition, investigation, methodology, supervision, writing – review and editing. **Georgina Oliver:** formal analysis, investigation, project administration, writing – review and editing. **Yen D. Y. Sim:** formal analysis, writing – review and editing. **Sabine Braat:** conceptualization, funding acquisition, methodology, visualization, writing – review and editing. **Georgia Cotter:** formal analysis, visualization, writing – review and editing. **Mary Lou Chatterton:** formal analysis, methodology, writing – review and editing. **Cathrine Mihalopoulos:** conceptualization, funding acquisition, methodology, writing – review and editing. **Sarah J. Wilson:** conceptualization, funding acquisition, methodology, supervision, writing – review and editing. **Trudie Chalder:** conceptualization, funding acquisition, methodology, supervision, writing – review and editing.

## Ethics Statement

The project received HREC approval from Austin Health HREC (project numbers: HREC/62334/Austin‐2020 and HREC/69382/Austin‐2021).

## Consent

Participants were required to provide written, informed consent on an HREC‐approved Participant Information and Consent Form (PICF) prior to commencing the studies (qualitative interviews and pilot study).

## Conflicts of Interest

The authors declare no conflicts of interest.

## Supporting information


**Supplementary Table 1:** Descriptions of Self‐Report Questionnaaires. **Supplementary Table 2:** Adverse Events Reported During Pilot Testing. **Supplementary Table 3:** Median of Self‐Report and Clinician‐Report Questionnaire Responses with Interquartile Range Across all Timepoints of Pilot Study. **Supplementary Table 4:** Resource Use Questionnaire Responses for Service Use at Baseline and Session 12.

## Data Availability

Data sharing is available for projects with approval from a registered HREC.
