# Supplementary material for: The Development and Acceptability of a Psychology‐Based Intervention for Debilitating Symptom Complexes Attributed to Ticks
Source: Health Expect. 2026 Mar 12;29(2):e70634. doi: 10.1111/hex.70634 (PMC13080887; doi:10.1111/hex.70634)
Supplement: Supplementary file 1 — Supplementary Table 1: Descriptions of Self‐Report Questionnaaires. Supplementary Table 2: Adverse Events Reported During Pilot Testing. Supplementary Table 3: Median of Self‐Report and Clinician‐Report Questionnaire Responses with Interquartile Range Across all Timepoints of Pilot Study. Supplementary Table 4: Resource Use Questionnaire Responses for Service Use at Baseline and Session 12. [file HEX-29-e70634-s001.docx]

**SUPPLEMENTARY MATERIAL FOR**

The Development and Acceptability of a Psychology-Based Intervention for Debilitating Symptom Complexes Attributed to Ticks

**This file contains:**

Supplementary Text 1-2

Supplementary Tables 1-4

**Supplementary Text 1**

*Brief Description of Two Common Cognitive-Behavioural Therapy Models Provided to Participants During Qualitative Interviews in Phase 1*

“I’d like to tell you now about a couple of treatment approaches we are considering for people with DSCATT to see what you think.

The first one is Cognitive behaviour therapy, or CBT for short. CBT is a talking therapy which helps people to see the connection between their symptoms, thoughts and behaviour (what they do).  By changing one of these a change is brought about in the others. So, in the case of DSCATT, symptoms will be affected by what people do and their perceptions of them. CBT focuses on changing behaviour and addressing thoughts that may be self-defeating to try to improve symptoms.

The second one is acceptance and commitment therapy, or ACT for short. This is a talking therapy which shows people how to avoid unhelpful thoughts, feelings and behaviours. It focuses on acceptance of things one cannot change and encourages people to live a valued life - irrespective of where they start. In DSCATT this would be no different. Unlike other approaches, it doesn’t try to reduce symptoms. It helps people to understand their approach to symptoms to see if it improves their quality of life or makes it worse. It is an approach which can improve people’s lives. Because it doesn’t depend on what causes symptoms, ACT enables people to accept what can’t be changed, while committing to what can be changed, in order to do what is important to them.

Both approaches involve behaviour change and help people to identify what’s important to them. The focus is on doing things despite the severity of symptoms. More often than not symptoms change with both approaches, and they have proven helpful for people with a wide range of diseases, disabilities or problems.

How would you feel about a course of therapy like CBT or ACT? Do they make sense to you? What are you thoughts regarding a treatment such as this delivered in person, versus via telehealth?”

**Supplementary Text 2DSCATT Pilot Study Exit Interview topic guide**Version 1 - 26 September 2020

**Introduction**

- Confirm identity and introduce interviewer
- Confirm willingness to participate in interview
- Remind of voice recording and confidentiality of interview and analysis
- Remind that interview is being voice recorded for transcription and analysis
- Explanation of objective of determining the acceptability of the treatment
- Structure and duration of the interview (free discussion 5-10 minutes, specific questions at the end for five minutes)
- **Any questions before we begin?** If there is any serious concern which cannot be satisfactorily resolved, offer for Prof Kanaan to call back before preceding to interview.

**Opening question:**

- **“I’d like to hear your views on the treatment you got from the DSCATT study based at Austin Health. Looking back on the sessions that you had, can you tell me a bit about what you thought of the treatment you received. Where should we start first?”**

**Targeted probes (when indicated):**

- If participant seems keen to talk about their current health or experiences and it doesn’t seem relevant to understanding their perspectives on the treatment, acknowledge what they are saying and then try to orient them back to talking about their views of the treatment itself, if necessary say, **“I can see that there’s a lot that you’d like to tell me, but because we only have a little while together, I’d like us to go back to talking about your experience of the treatment”**
- Encourage the participant to talk freely about the treatment without suggesting topics. Listen for reference to components of the study (see appendix) and when appropriate, prompt to explore them one by one by saying: **“You mentioned ‘X’ what was that like? Can you tell me more about it?”**
- When the participant is not generating any more information in response to the above probes or time is nearly up: **“we’re coming to the end of the free discussion part of the interview, and I just wanted to check, is there anything else that we haven’t talked about, or which you wanted to say something more about before we go on to the more specific questions?**

**General probes (when appropriate):**

- **I’d like to hear what you liked about the treatment and also what you didn’t like.**
- **You mentioned some ’good/bad’ things, was there anything ‘bad/good’?**
- **Can you tell me more about that? / Can you remember anything else?**
- **You mentioned that ‘X’ was ‘good/bad’ what do you think made it like that?**
- **It sounds like you’re saying that ‘X’ was significant, can you tell me more about ‘X’?**
- **Was there anything else about the experience that you can tell me about?**
- **Was there anything specific that you remember about it?**

**Specific Questions:**

1. Overall how would you rate your experience of the treatment you got at the DSCATT Pilot Study?

**Bad, fair, good, excellent**

1. Do you feel that you have benefited from this treatment in terms of your **physical** health?

**It made me feel worse, I didn’t really benefit at all, I benefitted a bit, I benefitted a lot**

1. Do you feel that you have benefited from the treatment in terms of your **emotional** wellbeing?

**It made me feel worse, I didn’t really benefit at all, I benefitted a bit, I benefitted a lot**

1. Do you feel that you have benefited from the treatment in terms of your **overall** health?

**It made me feel worse, I didn’t really benefit at all, I benefitted a bit, I benefitted a lot**

1. Do you feel that you have benefited from the treatment in terms of your **understanding** of DSCATT?

**Definitely not, probably not, probably have, definitely have**

1. Would you recommend a treatment like the one you had to someone who was suffering from DSCATT?

**Definitely not, probably not, probably would, definitely would.**

**Ending**

- Thank participant for their time and information.
- Any other questions? Answer if possible or offer for therapist to call them back to answer these questions if not able to answer.

**Supplementary Table 1**

*Descriptions of Self-Report Questionnaires*

| **Questionnaire** | **Description** |
| --- | --- |
| The Assessment of Quality of Life (AQoL)-8D^a^ | A multi-attribute utility self-report measure of health-related quality of life over the past week. It is comprised of 35-items grouped into 8 separately scored dimensions, which are summed to deriving either a psychometric score or a global utility score. Higher scores indicate lower quality of life. |
| Cognitive and Behavioural Responses to Symptoms Questionnaire Short (CBRQ-S)^b^ | An 18-item self-report questionnaire consisting of six subscales. Items are scored on a 5-point Likert scale from 0 (*Strongly disagree*) to 4 (*Strongly agree*), with a higher subscale score indicating increased presence of unhelpful cognitive or behavioural responses. |
| The Comprehensive assessment of Acceptance and Commitment Therapy processes (CompACT)^c^ | A 23-item self-report measure of psychological flexibility as conceptualized in ACT. Responses are given on a 7-point Likert scale from 0 (*Strongly disagree)* to 6 (*Strongly agree*) and scored via a scoring key to produce an overall psychological flexibility score as well as 3 subscale scores for different aspects of psychological flexibility: Openness to Experience, Behavioural Awareness, Valued Action. Higher scores indicate greater psychological flexibility. |
| Hospital Anxiety and Depression Scale (HADS)^d^ | A self-report measure of anxiety and depression over the past week consisting of 14 items (7 items for each subscale). Items are scored on a 4-point Likert scale ranging from 0 to 3 and summed within each subscale to produce an anxiety score (HADS-A) and a depression score (HADS-D) ranging from 0-21, where higher scores indicate higher distress. |
| Horowitz Lyme-MSIDS Questionnaire (HMQ)^e^ | A self-report questionnaire designed to determine the probability of the presence of Lyme Disease or another tick-borne disorder. Higher scores indicate higher probability of disease. Section 1 asks participants to rate the frequency of 38 symptoms on a scale of frequency from 0 (none) to 3 (all of the time), where scores are summed to produce a Symptom Frequency Score.  Section 2 contains 10 Yes/No items related to the likelihood of having Lyme disease, where a Yes-rated response yields a varying number of points. Scores are summed to produce a Lyme Incidence Scale Score ranging from 0-34.  Section 3 contains 2 items asking how many days in the past 30 days were not good in terms of overall physical health and mental health, respectively. These two questions are scored as follows: 0–5 days = one point; 6–12 days = two points; 13–20 days = three points; 21–30 days = four points. |
| Illness Perception Questionnaire – Revised (IPQR)^f^ | A self-report measure of participants’ perceptions of their illness and treatment divided into three sections. Section 1 asks participants to rate whether they have experienced a list of 12 symptoms using a Yes/No response, and then asks whether they believe each symptom to be related to their illness using a Yes/No response. Yes-rated items from this second question are summed to create the Illness Identity subscale.  Section 2 asks the participant to rate their agreement with 38 statements about their illness on a 5-point Likert scale from 1 (*Strongly* *disagree*) to 5 (*Strongly* *agree*). Respective items are summed to derive seven separate subscale scores (Consequences, Timeline [Acute/Chronic], Timeline [Cyclical], Personal Control, Treatment Control, Illness Coherence, and Emotional Representations).  Section 3 asks the participant about their beliefs surrounding the cause of their illness, where they rate 18 attributional items on a 5-point Likert scale from 1 (*Strongly disagree*) to 5 (*Strongly agree*).  Higher scores indicate more negative perceptions and beliefs about the illness, except for the Personal Control and Treatment Control subscales. |
| Patient Global Impressions severity (PGI-S) and improvement (PGI-I) scales^g^ | Single-item self-report measures of symptom severity and improvement, respectively. The PGI-S requires participants to rate their symptom severity on a 4-point scale (*Absent, Mild, Moderate, Severe*). The PGI-I asks participants to rate how their symptoms have changed since baseline on a 7-point scale from 1 (*Very much better*) to 7 (*Very much worse*). Higher scores indicate greater severity and less improvement. |
| Resource Use Questionnaire (RUQ; Supplementary Table 3) | Adapted by health economists within the research team (CM, M-LC) from previous Australian trials of mental health interventions. It is a self-report measure of healthcare resource/service use, lost paid and unpaid work (absenteeism), and productivity while at work with symptoms (presenteeism). |
| Work and Social Adjustment Scale (WSAS)^h^ | A 5-item self-report scale that measures functional impairment attributable to a specific illness, where responses to statements are rated on a 9-point Likert scale from 0 (*Not at all*) to 8 (*Very severely*). Items are summed to derive a total score ranging from 0 to 40, with higher scores indicating greater functional impairment. |

*Note*.

^a^ Richardson J, Iezzi A, Khan MA, Maxwell A. Validity and reliability of the Assessment of Quality of Life (AQoL)-8D multi-attribute utility instrument. *Patient*. 2014;7(1):85-96.

^b^ Devilly GJ, Borkovec TD. Psychometric properties of the credibility/expectancy questionnaire. *J Behav Ther Exp Psychiatry*. 2000/06/01/ 2000;31(2):73-86.

^c^ Ryan EG, Vitoratou S, Goldsmith KA, Chalder T. Psychometric Properties and Factor Structure of a Long and Shortened Version of the Cognitive and Behavioural Responses Questionnaire. *Psychosom Med*. Feb/Mar 2018;80(2):230-237.

^d^ Francis AW, Dawson DL, Golijani-Moghaddam N. The development and validation of the Comprehensive assessment of Acceptance and Commitment Therapy processes (CompACT). *J Contextual Behav Sci*. 2016/07/01/ 2016;5(3):134-145.

^e^ Zigmond AS, Snaith RP. The hospital anxiety and depression scale. *Acta Psychiatr Scand*. Jun 1983;67(6):361-70.

^f^ Citera M, Freeman PR, Horowitz RI. Empirical validation of the Horowitz Multiple Systemic Infectious Disease Syndrome Questionnaire for suspected Lyme disease. *Int J Gen Med*. 2017;10:249-273.

^g^ Moss-Morris R, Weinman J, Petrie K, Horne R, Cameron L, Buick D. The Revised Illness Perception Questionnaire (IPQ-R). *Psychol Health*. 2002/01/01 2002;17(1):1-16.

^h^ Guy W. *ECDEU Assessment Manual for Psychopharmacology*. US Department of Heath, Education, and Welfare - Public Health Service Alcohol, Drug Abuse, and Mental Health Administration; 1976.

^i^ Mundt JC, Marks IM, Shear MK, Greist JH. The Work and Social Adjustment Scale: a simple measure of impairment in functioning. *Br J Psychiatry*. May 2002;180:461-4.

**Supplementary Table 2**

*Adverse Events Reported During Pilot Testing.*

| **Adverse Event** | **Severity** | **Causality to Intervention** |
| --- | --- | --- |
| Shortness of breath, faster breathing and a "crushing" feeling in chest | Moderate | Unrelated |
| Delayed gut motility | Severe | Unrelated |
| Irritability | Unknown/not assessed | Possibly |
| Back and abdominal pain, subsequent bilateral hernia diagnosis | Moderate | Unrelated |
| Vertigo^‡^ | Severe | Unlikely |
| Blackout | Severe | Unrelated |
| Diverticulosis diagnosis | Unknown/not assessed | Unrelated |
| Fall when bike riding | Moderate | Unrelated |
| Pain behind left eye | Severe | Unlikely |
| COVID infection | Mild | Unrelated |
| Chest and rib pain | Unknown/not assessed | Unrelated |
| Tremor (hands) with concurrent nodding of head^‡^ | Mild | Unlikely |
| Swollen hands and feet^‡^ | Moderate | Unrelated |
| Speech difficulties^‡^ | Moderate | Unrelated |
| Foot pain^‡^ | Moderate | Unlikely |
| "Flu-like symptoms" | Moderate | Unlikely |
| Vomiting at night and dry retching in mornings^‡^ | Unknown/not assessed | Unlikely |
| Hospitalisation for FND^†^ | Severe | Unlikely |
| Low mood with suicidal ideation | Moderate | Unlikely |
| Self-harming urges | Moderate | Possibly |

*Note.* ^†^Constituted a Serious Adverse Event. ^‡^Reported by participant to be a re-emerging symptom in the context of their DSCATT illness.

FND = Functional Neurological Disorder

**Supplementary Table 3**

*Median of Self-Report and Clinician-Report Questionnaire Responses with Interquartile Range Across all Timepoints of Pilot Study*

| **Questionnaire** | **Baseline**  n = 7 | **Session 4**  n = 6 | **Session 8**  n = 5 | **Session 12**  n = 6 |
| --- | --- | --- | --- | --- |
| **PGI-S ^a^** | 3.00 (3.00-3.00) | 3.00 (2.00-3.00) | 3.00 (2.50-3.50) | 3.00 (2.00-3.25) |
| **PGI-I ^a^** | - | 4.00 (3.75-6.00) | 5.00 (4.00-6.00) | 3.50 (2.75-6.25) |
| **CompACT^b^** |  |  |  |  |
| Total Score | 92.00 (77.00-107.00) | - | - | 91.00 (80.25-110.75) |
| Openness to Experience | 39.00 (26.00-45.00) | - | - | 36.00 (27.75-46.25) |
| Behavioural Awareness | 18.00 (13.00-23.00) | - | - | 18.50 (13.75-21.75) |
| Valued Action | 38.00 (33.00-41.00) | - | - | 40.00 (34.00-42.25) |
| **AQoL-8D^a^** |  |  |  |  |
| Total score | 65.25 (60.28-70.92) | 55.32 (48.05-69.86) | 65.96 (45.75-71.99) | 62.06 (40.25-68.09) |
| Independent Living | 61.11 (44.44-77.78) | 52.78 (38.89-59.73) | 44.44 (36.11-58.34) | 61.12 (50.00-68.06) |
| Happiness | 68.75 (50.00-68.75) | 53.13 (45.31-68.75) | 75.00 (31.25-75.00) | 53.13 (31.25-70.31) |
| Mental Health | 72.73 (51.52-72.73) | 53.04 (43.18-81.82) | 75.76 (57.58–80.31) | 59.09 (35.60-81.82) |
| Coping | 58.33 (41.67-66.67) | 50.00 (35.42-60.42) | 33.33 (25.00-66.67) | 50.00 (31.25-66.67) |
| Relationships | 70.37 (66.67-77.78) | 62.96 (56.48-76.85) | 66.67 (50.00-74.07) | 59.26 (47.22-67.60) |
| Self-worth | 75.00 (66.67-83.33) | 66.67 (39.59-75.00) | 66.67 (25.00-87.50) | 66.67 (29.17-75.00) |
| Pain | 40.00 (20.00-50.00) | 35.00 (25.00-62.50) | 40.00 (30.00-90.00) | 40.00 (30.00-52.50) |
| Senses | 76. 92 (69.23-84.62) | 73.08 (67.31-76.92) | 69.23 (61.54-84.62) | 76.92 (71.15-84.62) |
| **CBRQ-S^a^** |  |  |  |  |
| All-or-Nothing Behaviour | 6.00 (2.00-10.00) | - | - | 5.00 (3.75-6.25) |
| Avoidance/Resting Behaviour | 5.00 (3.00-9.00) | - | - | 4.00 (2.25-5.25) |
| Fear Avoidance | 7.00 (4.00-7.00) | - | - | 6.5.0 (5.00-7.13) |
| Embarrassment Avoidance | 6.00 (3.00-9.00) | - | - | 6.50 (1.75-10.25) |
| Symptom-Focusing | 7.00 (6.00-8.00) | - | - | 8.00 (6.50-9.25) |
| Damage Beliefs | 9.00 (6.00-9.00) | - | - | 7.00 (5.75-8.00) |
| **HMQ^a^** |  |  |  |  |
| Symptom Frequency Score | 52.00 (36.00-55.00) | 44.13 (42.08-46.75) | 41.00 (31.50-47.16) | 44.04 (36.52-51.00) |
| **WSAS^a^** | 24.00 (18.00-29.00) | 31.00 (23.75-34.00) | 24.00 (20.00-35.00) | 28.00 (17.25-35.75) |
| **HADS^a^** |  |  |  |  |
| Depression Score | 8.00 (5.00-8.00) | 9.00 (6.50-10.50) | 7.00 (3.00-11.50) | 7.00 (4.75-10.75) |
| Anxiety Score | 6.00 (5.00-10.00) | 7.50 (3.25-8.75) | 4.00 (2.00-9.50) | 5.50 (1.50-10.00) |
| **IPQ-R^a^** |  |  |  |  |
| Illness Identity Score | 10.00 (9.00-13.00) | - | - | - |
| Timeline (acute/chronic) | 20.00 (17.00-21.00) | - | - | - |
| Timeline (cyclical) | 17.00 (16.00-18.00) | - | - | - |
| Consequences | 24.00 (22.00-27.00) | - | - | - |
| Personal control^b^ | 20.00 (17.00-24.00) | - | - | - |
| Treatment control^b^ | 17.00 (15.00-21.00) | - | - | - |
| Illness coherence | 15.00 (10.00-16.00) | - | - | - |
| Emotional representations | 20.00 (16.00-23.00) | - | - | - |
| *Causal Dimensions* |  | - | - | - |
| Psychological Attributions | 10.50 (6.00-19.25) ^†^ | - | - | - |
| Risk Factors | 10.50 (8.00-15.00) ^†^ | - | - | - |
| Immunity | 10.50 (6.00-11.00) ^†^ | - | - | - |
| Accident or Chance | 4.00 (2.00-6.00) ^†^ | - | - | - |
| **CGI^a^** |  |  |  |  |
| Severity | 5.00 (4.00-5.00) | - | - | 5.00 (3.50-5.25)^c^ |
| Improvement | - | - | - | 3.50 (2.75–4.50) ^c^ |

*Note.* ^†^n = 6 at Baseline due to missing data (participant non-response).

^a^Higher scores on this measure indicate worse outcomes

^b^Higher scores on this measure indicate better outcomes

^c^CGI was completed by the project coordinator at Baseline and after the Exit Interview

AQoL-8D = The Assessment of Quality of Life 8D; CBRQ-S = Cognitive and Behavioural Responses to Symptoms Questionnaire; CGI = Clinical Global Impressions Scale; CompACT = Comprehensive assessment of Acceptance and Commitment Therapy processes; HADS = Hospital Anxiety and Depression Scale; HMQ = Horowitz Lyme-MSIDS Questionnaire; IPQR = Illness Perception Questionnaire – Revised; PGI-I = Patient Global Impressions improvement scale; PGI-S = Patient Global Impressions severity scale; WSAS = Work and Social Adjustment Scale

**Supplementary Table 4**

*Resource Use Questionnaire Responses for Service Use at Baseline and Session 12.*

|  | **Baseline (N = 7)** | | **Session 12 (N = 6)** | |
| --- | --- | --- | --- | --- |
|  | **n** | **%** | **n** | **%** |
| **Health Service Use** |  |  |  |  |
| General Practitioner | 7 | 100 | 6 | 100 |
| Specialist Doctor | 5 | 71 | 5 | 83 |
| Hospital Doctor | 2 | 29 | 3 | 50 |
| Lyme-Literate Doctor | 2 | 29 | 1 | 17 |
| Psychologist | 2 | 29 | 2 | 33 |
| Psychiatrist | 1 | 14 | 0 | 0 |
| Counsellor | 0 | 0 | 0 | 0 |
| Physiotherapist | 1 | 14 | 3 | 50 |
| Chiropractor | 3 | 43 | 2 | 33 |
| Social Worker | 0 | 0 | 2 | 33 |
| Alcohol or Drug Worker | 0 | 0 | 0 | 0 |
| Support Group | 0 | 0 | 0 | 0 |
| Family Therapist | 0 | 0 | 0 | 0 |
| Naturopath | 1 | 14 | 0 | 0 |
| Complementary/Alternative Therapist | 2 | 29 | 0 | 0 |
| Pharmacist | 2 | 29 | 3 | 50 |
| Other | 5 | 71 | 2 | 33 |
| **Diagnostic Tests** | 6 | 86 | 5 | 83 |
| **Hospital Attendance & Ambulance Use** |  |  |  |  |
| Emergency Department | 2 | 29 | 4 | 67 |
| Hospital/Day Surgery/Care Facility | 2 | 29 | 1 | 17 |
| Respite/Residential Care/Rehabilitation | 0 | 0 | 0 | 0 |
| Ambulance | 0 | 0 | 2 | 33 |
| **Medications** |  |  |  |  |
| Prescription Medications | 5 | 71 | 5 | 83 |
| Vitamins/Supplements | 5 | 71 | 5 | 83 |
| **Other Services** |  |  |  |  |
| Financial Advice | 1 | 14 | 1 | 17 |
| Housing Advice | 0 | 0 | 0 | 0 |
| Vocational Guidance | 0 | 0 | 0 | 0 |
| Secure Welfare Services | 0 | 0 | 0 | 0 |
| Legal Advice | 1 | 14 | 0 | 0 |
| Independent Living Skills | 0 | 0 | 0 | 0 |
| Other | 0 | 0 | 0 | 0 |
| **School and Work** |  |  |  |  |
| Missed School | 0^†^ | 0^†^ | 0^¶^ | 0^¶^ |
| Missed Paid Work | 4^‡^ | 80^‡^ | 3^§^ | 100^§^ |
| Missed Unpaid Work | 3^‡^ | 60^‡^ | 3^‡^ | 60^‡^ |
| Presenteeism | 5^‡^ | 100^‡^ | 2^†^ | 50^†^ |

*Note.* ^†^n = 4 at this timepoint due to missing participant response; ^‡^n = 5 at this timepoint due to missing participant responses; ^§^n = 3 at this timepoint due to missing participant responses; ^¶^n = 1 at this timepoint due to missing participant responses.
